# Access to tobacco cessation is a human right and essential for the endgame

**DOI:** 10.18332/tid/203618

**Published:** 2025-05-06

**Authors:** Ashton Thiele, Carolyn Dresler, Hasmeena Kathuria, Megan Arendt-Manning, Carol Southard

**Affiliations:** 1 Action on Smoking and Health, Washington, United States; 2 Center for Tobacco Research and Intervention, University of Wisconsin, Madison, United States; 3 Tobacco Treatment Department, Endeavor Health Services Clinic Treatment, New York, United States

**Keywords:** cessation, equity, endgame, prevention

The tobacco epidemic ravages communities around the world, and most people who smoke cigarettes want to quit (around 70% of all tobacco users)^1^. Providing tobacco treatment is an essential tool for clinics, hospitals, and governments in preventing or decreasing mortality from tobacco-induced diseases. Preventing tobacco addiction and providing assistance in quitting are key to protecting the human right to health. However, there remains a problem. Less than 4 in every 10 people who smoke are offered cessation support by their healthcare provider^2^. Most people attempt to quit smoking without expert assistance, but this method has the lowest success rate, with only 4–6% of people successfully quitting tobacco on their own^2^. Given these challenges, healthcare providers and advocates must promote cessation support and provide universal access to cessation treatments to prevent and/or mitigate the harm that tobacco smoking inflicts on its victims.

Access to comprehensive cessation support has taken on a new urgency in light of the so-called ‘tobacco endgame’ movement, which seeks to end rather than mitigate the tobacco epidemic. Twenty-one municipalities in the US have already passed endgame bills, and they are being seriously considered in the United Kingdom and South Australia. While these laws are often touted as a way to protect future generations from tobacco addiction, disease and death, human rights obligations demand that any government passing endgame must ensure that existing adults who smoke are not left behind.

The effectiveness of comprehensive cessation support is well documented. Simply put, practitioners in clinical settings are not doing enough to provide tobacco cessation treatment as only 63% of patients were screened for tobacco use; around 21% received cessation counseling, and 8% were provided cessation medication [varenicline, cytisine, nicotine replacement therapy (NRT), bupropion]^3^. Furthermore, only 5% of people who smoke received both counseling and medication to quit, even though combination therapy is more effective than either method alone^4^.

Action on Smoking and Health, Endeavor Health, and the University of Wisconsin Center for Tobacco Research and Intervention (UW-CTRI) hosted a webinar on 30 January 2025, to hear from experts in the field on how to improve tobacco cessation treatment access. The event recording is available here . A new fact sheet was also developed to present: How Advocates and Policymakers can Bolster Tobacco Cessation here>

The first speaker at the webinar was C. Southard, a specialist on tobacco control and a passionate advocate for cessation support, who has dedicated her career to providing tobacco cessation support for close to 40 years. She provided an overview of the 2008 Public Health Service (PHS) tobacco treatment guidelines and discussed the importance of including and prioritizing evidence-based tobacco cessation interventions in tobacco control efforts^5^. She highlighted that while tobacco treatment interventions (counseling and pharmacotherapy) are effective, they are grossly underutilized. She advocated that tobacco treatment should be included in all clinical encounters on a regular basis. As an example, C. Southard outlined the components of an 8-week evidence-based clinical program that she implemented. She has documented quit rates since its inception and discussed the positive results in smoking cessation outcomes with such a program. Thus, she has found that because most people who smoke want to quit, with a dedicated and trained tobacco treatment expert, the chance of success can be high.

C. Southard also shared the relevant CPT (Current Procedural Terminology) codes for tobacco cessation counseling: CPT code 99406 is used for an intermediate counseling visit lasting more than 3 minutes but up to 10 minutes, while CPT code 99407 is for an intensive counseling visit lasting more than 10 minutes. These codes help healthcare providers document and bill for the time and effort spent helping patients quit smoking, which may encourage healthcare providers to integrate these services into their clinical practice^6^.

The next speaker on the webinar was H. Kathuria, Director of the University of Wisconsin’s Center for Tobacco Treatment and Intervention (UW-CTRI). She discussed the importance of integrating smoking cessation interventions and clinical decision support tools into electronic health records (EHRs). Additionally, she emphasized the importance of increasing access to tobacco treatment with tobacco control policy changes, such as banning menthol as a characterizing flavor in cigarettes and setting nicotine standards in cigarettes to minimally addictive or non-addictive levels.

EHR integration into health systems can help clinicians easily access resources during patient care and prompt them to offer cessation counseling and treatment. H. Kathuria highlighted several methods to achieve this integration, such as incorporating cessation services into the lung cancer continuum and using opt-out approaches to increase the reach of tobacco treatment to people who smoke. Opt-out approaches involve automatically delivering tobacco treatment to individuals who smoke, regardless of their readiness to quit or clinical diagnosis, unless they decline treatment.

As an example of an opt-out cessation service, H. Kathuria described an approach she implemented at a large safety-net hospital that proactively treats all patients who are admitted to the hospital and use tobacco products. The opt-out cessation service uses the EHR to identify individuals who use tobacco. Patients then receive opt-out support by a tobacco treatment consultation (TTC) service that delivers, bedside counseling, recommendations for pharmacotherapy while hospitalized and a linkage to outpatient treatment at discharge. This approach has proven to be sustainable and highly effective, as individuals seen by the TTC service had higher smoking cessation rates and a higher likelihood of receiving nicotine replacement treatment compared to those not seen by the service^7,8^.

Strengthening health policy is also crucial to integrating cessation into routine healthcare services and helps lessen the tobacco use epidemic. In recognizing and accepting that access to evidence-based cessation therapy is key to the right to health, we must also recognize that governments have a duty to provide such support. Tobacco use is a major cause of the two leading causes of death in the Black community: cancer and heart disease. Black individuals are also much less likely to be screened for lung cancer despite being eligible candidates^9^. In addition, Black and Hispanic lifetime cigarette users are much less likely to undergo cessation treatment, such as nicotine replacement therapy (NRT). Around 22% of Latino and Black community members received such therapies, compared to 31% of White community members. Cessation support must be provided equally to eliminate racial disparities^10^. Barriers to care make cessation more difficult for minority communities, amplifying the need to ensure equitable access to tobacco cessation treatments, including broad support for policies, such as the menthol ban^11^.

Furthermore, tobacco companies have marketed menthol cigarettes to minority communities, co-opting their culture and icons in advertisements. One study has shown that across seven provinces of Canada, people who smoke menthol cigarettes were less likely to successfully quit than individuals who smoke non-menthol cigarettes^12^. Robust public health policy implementation, like menthol bans and equitable access to expert, evidence-based tobacco cessation care, can save millions of lives. Access to the human right to health is achieved through proactive health policy changes and equitable access to effective tobacco cessation treatment, resulting in a healthier society. Federal cessation policies, inclusive of good cessation programs in all healthcare facilities, can accelerate the end of the tobacco epidemic.

## Data Availability

Data sharing is not applicable to this article as no new data were created.
